# Diagnosis Concordance in Liaison Psychiatry Patients Transferred From a tertiary Center to Inpatient Psychiatric Care

**DOI:** 10.1192/bjo.2025.10697

**Published:** 2025-06-20

**Authors:** Tabea Winkler, Nadine Cossette

**Affiliations:** Royal Infirmary of Edinburgh, Edinburgh, United Kingdom

## Abstract

**Aims:** A subset of patients assessed by the Liaison Psychiatry service at the Royal Infirmary of Edinburgh are transferred for inpatient psychiatric care. The aim of this audit was to investigate diagnosis concordance, length of stay and nature of follow-up in this cohort. A comparison was made with a previous version of this audit from 2019.

**Methods:** A review of the relevant cohort took place using internally recorded data from the liaison psychiatry service and inpatient discharge letters from Trak (an electronic notes system). The chosen time period spanned 01/01/24–28/06/24 (n=68). Patients were excluded if no clear working diagnosis was available, they were admitted to an inpatient facility not using Trak or if they were transferred from and subsequently returned to IP care (n=54). Diagnosis concordance was split into complete agreement/match to disorder/match to group of disorder/match with +/- 1 additional diagnosis/no match.

**Results:** Demographic overview: 82% of patients had been discharged from IP care by the end of the audited time period. 55% of transferred patients were male; 45% female. Patients were most commonly aged between 31–35.

Length of stay: Length of stay ranged from 1–260 days, with a mean of 65.82 and a median of 36 days.

Diagnosis concordance: 33% had complete agreement, 8% match to disorder, 26% match to group of disorders, 20% had a match +/- another diagnosis and 13% had no match. Therefore, 87% of patients had a match of some kind. The most common diagnosis group was a mood disorder, followed by neurocognitive disorders and primary psychotic disorders.

Follow up: 44% had mixed follow up (>1 discipline), 24% CMHT, 7% IHTT, 9% RRT, 7% CPN, 7% specialist and 2% solely primary care.

**Conclusion:** In a majority of patients there was an element of diagnosis concordance. Liaison psychiatry diagnoses can partly be a snapshot based on a shorter stay, and inpatient admission may allow further details to come to light influencing diagnosis (i.e. first presentation psychosis to schizophrenia). Notably, in comparison to the 2019 median audit IP length of stay had increased by 11 days. Hypotheses explaining this include a changing patient cohort overall or increased bed pressures leading to a different subsection of patients being admitted to IP care. The most common disorder group (mood disorder) is in line with a high percentage of patients presenting to the RIE secondary to intentional overdose with suicidal intent.

